# A biomaterials roadmap from retinoic acid biology to kidney regenerative therapy

**DOI:** 10.1016/j.isci.2026.117554

**Published:** 2026-09-18

**Authors:** Ru-Yu Tan, Li-Li Huang, Qin-Ying She, Yun-Ci Ma, Min-Hong Liu, Li-Juan Li, Chao Zhang, Yi-Wen Zhong, Shan Zhou, Hui-Xin Bi

**Affiliations:** 1 Department of Nephrology, The Fifth Affiliated Hospital, Southern Medical University, Guangzhou 510900, China; 2 State Key Laboratory of Organ Failure Research, National Clinical Research Center of Kidney Disease, Guangdong Provincial Clinical Research Center for Kidney Disease, Guangdong Provincial Key Laboratory of Nephrology, Division of Nephrology, Nanfang Hospital, Southern Medical University, Guangzhou 510515, China; 3 Sichuan Clinical Research Center for Nephropathy, Chengdu 610000, China

**Keywords:** retinoic acid, renal regeneration, biomaterials, nanocarriers, multifunctional scaffolds, developmental bioinspiration

## Abstract

The mammalian kidney cannot generate new nephrons after birth, a key driver of chronic kidney disease progression toward end-stage renal disease. Retinoic acid (RA) is an evolutionarily conserved morphogen essential for kidney development, and its signaling is reactivated after injury to coordinate repair—yet this endogenous repair program is transient and insufficient to guide the kidney along the regenerative continuum toward *de novo* nephron formation. Despite its regenerative potential, RA’s clinical translation is limited by systemic toxicity, a narrow therapeutic window, and poor pharmacokinetics. Here, we synthesize evidence from developmental biology and injury models showing that RA can activate endogenous renal progenitor programs. We then analyze its pharmacological and toxicity barriers and introduce a developmental bioinspiration framework using renal-targeted nanocarriers and multifunctional scaffolds to overcome these obstacles. By recapitulating native RA signaling dynamics, these platforms aim to steer the adult kidney away from maladaptive fibrosis and toward functional restoration.

## Graphical abstract

**Figure:**
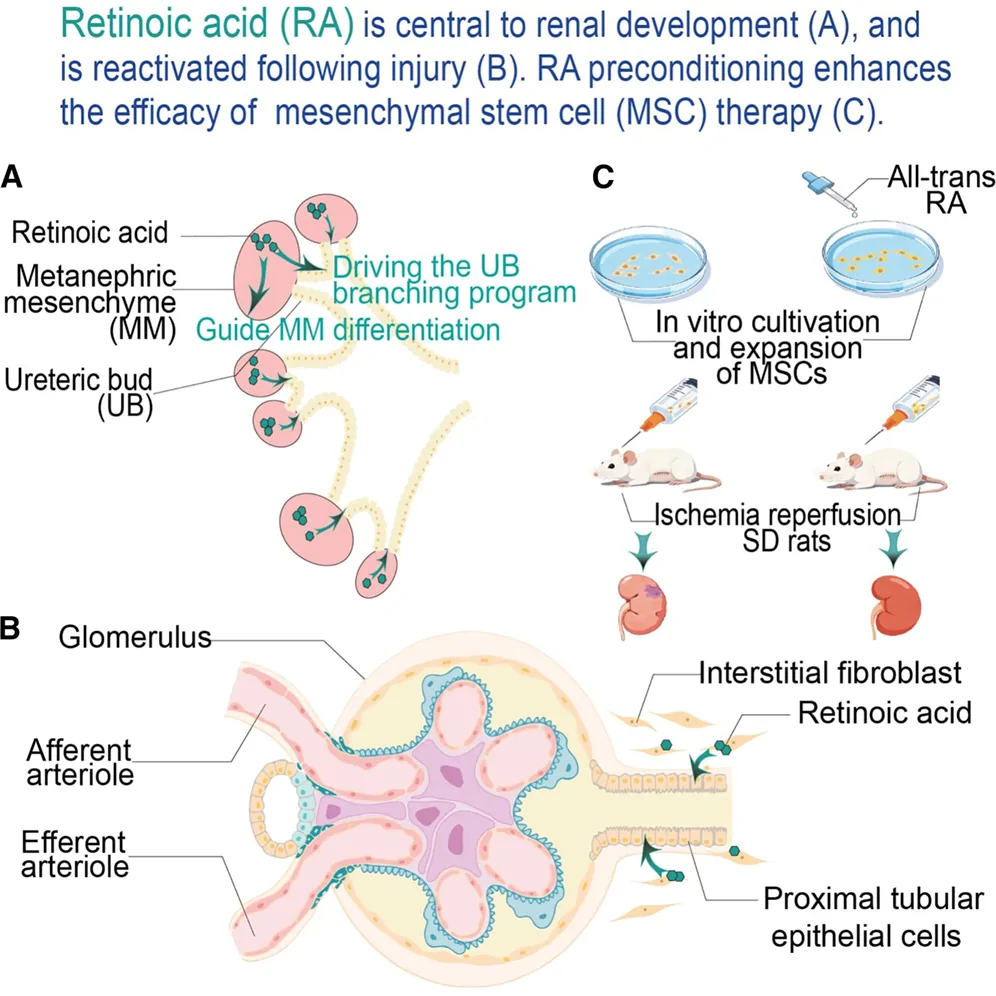

## Teaser

Therapeutics; Developmental biology; Biomaterials

## Introduction

The kidney is a vital organ responsible for excretion, fluid and electrolyte homeostasis, and endocrine functions. However, unlike certain vertebrates such as the axolotl (*Ambystoma mexicanum*), which can mount a complete regenerative response culminating in *de novo* nephron formation after injury,^1^ the adult mammalian kidney lacks this capacity. In mammals, upon injury, surviving tubular epithelial cells initiate a repair program characterized by dedifferentiation, proliferation, and subsequent redifferentiation—an adaptive response that represents an essential phase on the initial path toward regeneration.^2^ However, this program is highly susceptible to derailment: persistent inflammation, oxidative stress, mitochondrial dysfunction, and fibrotic signals often divert the endogenous repair program away from successful completion toward maladaptive repair—a trajectory characterized by failed redifferentiation, sustained cell-cycle arrest, and progressive fibrosis.^3^^,^^4^^,^^5^ By contrast, *de novo* nephron regeneration—the full completion of this program, culminating in the formation of entirely new nephrons—is achieved in axolotls but is essentially absent in adult mammals. This maladaptive outcome, rather than true regeneration, is what drives the transition from acute kidney injury (AKI) to chronic kidney disease (CKD). Thus, the goal of regenerative strategies is to guide this intrinsic program through its entire trajectory, steering the mammalian kidney as far as possible along the regenerative continuum.

Retinoic acid (RA), the principal bioactive metabolite of vitamin A, is a quintessential morphogen that governs a wide array of developmental and physiological processes.^6^^,^^7^ Synthesized from retinol via two sequential oxidative steps catalyzed by aldehyde dehydrogenase 1 family member A (ALDH1A) enzymes, RA signals through its nuclear receptors (RARα, RARβ, and RARγ) to regulate the transcription of numerous target genes, with its spatiotemporal activity tightly controlled by the balance between ALDH1A-mediated synthesis and CYP26-mediated degradation.^6^^,^^7^ During kidney development, RA plays a crucial role by regulating ureteric bud (UB) branching and nephron progenitor differentiation.^8^^,^^9^^,^^10^ Critically, this developmental role is not confined to embryogenesis: after acute kidney injury, RA signaling is re-engaged in proximal tubular epithelial cells (PTECs), promoting dedifferentiation and proliferation—a reactivation of embryonic programs that mirrors the early phases of the regenerative continuum previously described.^11^^,^^12^ This observation underscores a pivotal insight in regenerative biology that tissue repair often involves the reactivation of embryonic programs.^13^^,^^14^ Recently, a landmark study demonstrated that reactivating RA synthesis via *Aldh1a2* overexpression restores full structural regeneration—including cartilage, nerve, and skin—in otherwise non-regenerating adult mouse ear wounds.^15^ Although this model is non-renal, it provides proof-of-principle that reactivating RA synthesis can trigger a regenerative response in a non-regenerating adult mammalian tissue. This finding strengthens the conceptual rationale for exploring whether RA-based strategies can guide the mammalian kidney along the regenerative continuum.

However, translating RA into a regenerative therapy faces formidable pharmacological hurdles. Its pleiotropic effects cause dose-limiting systemic toxicity (e.g., hepatotoxicity and teratogenicity),^16^^,^^17^^,^^18^ while inherent properties like poor solubility, photosensitivity, and rapid metabolism hinder clinical utility.^19^^,^^20^^,^^21^ Furthermore, RA signaling exhibits a context-dependent “yin-yang” duality in the kidney, mediating both protective regeneration and pathogenic fibrosis or apoptosis.^22^ This narrow therapeutic window renders simple systemic administration ineffective and potentially harmful.

Thus, the clinical translation of RA for kidney regeneration demands a precision delivery strategy that can achieve spatiotemporally controlled and pulsed release at the injury site—thereby avoiding systemic toxicity, bypassing rapid metabolism, and recapitulating developmental signaling patterns. This review synthesizes evidence on RA’s dual roles in kidney pathology, identifies key pharmacological barriers, and evaluates biomaterial-based solutions (nanocarriers and scaffolds). We further propose a “developmental bioinspiration” paradigm that uses these platforms to mimic embryonic RA dynamics, aiming to shift the adult kidney’s response from fibrosis toward genuine regeneration.

## Literature search strategy

To evaluate the evidence for RA’s intrinsic kidney regenerative potential and to explore how biomaterial strategies might overcome its pharmacological barriers, we performed a targeted literature search in PubMed and Web of Science Core Collection (2000–2025). The search combined terms related to three pillars: (1) RA in kidney development and repair; (2) pharmacokinetic and toxicity barriers; and (3) advanced drug delivery systems. Boolean operators (AND, OR) were used to cross-link these domains.

## From construction to repair: Repurposing RA signaling from renal development to post-injury regeneration

### Developmental blueprint: RA orchestrates nephrogenesis

To understand how RA might promote renal regeneration, it is instructive to first examine its role in kidney development. In the developing kidney, nephron formation is driven by reciprocal interactions between the metanephric mesenchyme (MM) and the UB. These two compartments exchange signals that coordinate branching morphogenesis and nephron progenitor differentiation.^23^ As shown in Figure 1, the UB undergoes repetitive branching to form the future collecting ducts, while the MM provides the progenitor “soil” for nephrogenesis.^24^ The RA signaling pathway is a key regulator of this process.^25^ Mechanistically, RA produced by the MM via *Aldh1a2* upregulates *Ret* expression in the UB, thereby driving branching morphogenesis.^24^ This local RA signaling is in turn supported by the transcription factor Wilms tumor 1 (WT1), which maintains *Aldh1a2* expression in the cortical stroma. The functional importance of this WT1-RA-RET axis is demonstrated by stromal-specific *Wt1* knockout mice: loss of *Wt1* leads to a transient reduction in *Aldh1a2* expression and diminished RA signaling in the stroma, which in turn reduces *Ret* expression in UB tips and impairs branching morphogenesis. Importantly, exogenous RA administration *in vitro* partially rescues these branching defects, confirming that *Wt1* sustains stromal RA signaling to ensure proper *Ret* expression and dynamics in the UB.^26^ Beyond branching, RA maintains mesenchymal progenitor competence and, in concert with factors like WNTs and BMP4, guides their differentiation into specific renal cell types.^25^^,^^27^ The downstream effects of RA are transduced through its nuclear receptors, RARα, RARβ, and RARγ. However, the specific roles of individual RAR subtypes in nephrogenesis remain incompletely defined. The best-characterized evidence comes from compound knockout studies showing that RARα and RARβ2 function redundantly in stromal cells to maintain *Ret* expression and support UB branching.^28^ The roles of RARγ in nephrogenesis remain largely unexplored.

**Figure 1:**
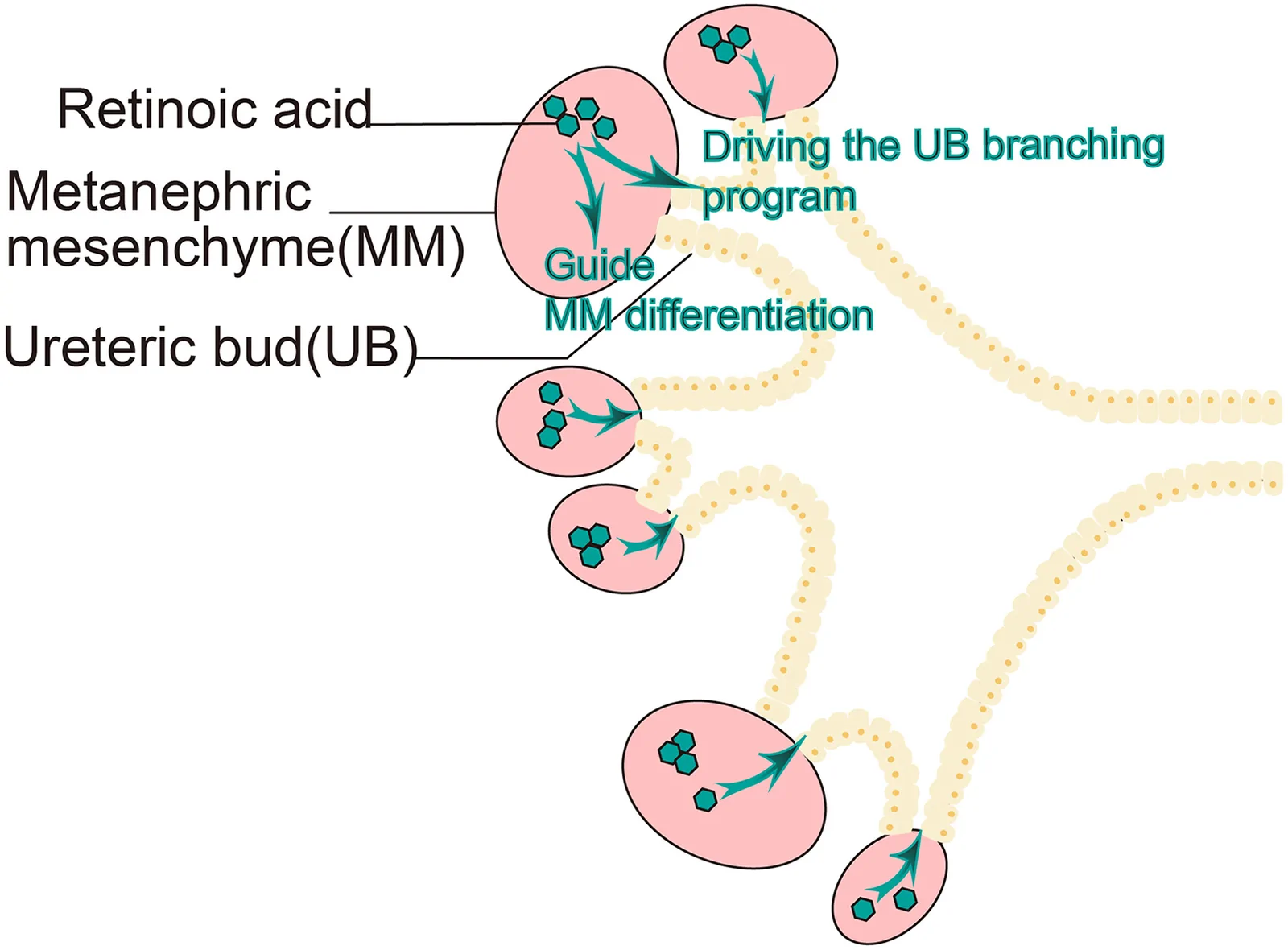
The role of RA in renal development: An evolutionarily conserved morphogen central to renal development RA coordinates kidney development by: (1) driving UB branching morphogenesis; and (2) maintaining nephron progenitor competence and guiding their differentiation. Abbreviations are as follows: RA, retinoic acid; UB, ureteric bud; MM, metanephric mesenchyme.

Further evidence for RA’s central role comes from kidney organoid models. The RA agonist TTNPB (a synthetic retinoic acid receptor agonist) is essential for inducing progenitor cells to form polarized renal vesicles, the foundational precursors to segmented nephrons.^29^ In contrast, RA supplementation at non-physiological levels can perturb the mesenchymal-epithelial balance, depleting nephron progenitor cells and altering nephron patterning.^30^ Therefore, RA signaling acts as a key hub coordinating mesenchymal-epithelial interactions and nephron production.

### Post-injury reactivation: Developmental programs replayed

The developmental role of RA is not confined to embryogenesis. In the adult kidney, although RA activity is maintained at a low baseline level and largely restricted to the collecting duct epithelia under physiological conditions,^8^ the kidney retains the capacity to readily reactivate RA signaling upon injury. In the adult kidney, injury—such as that seen in human sepsis or murine ischemia-reperfusion models—reinduces RA signaling in PTECs.^12^ This reparative response shares key features with developmental programs. At the cellular level, injured PTECs undergo dedifferentiation, proliferation, and redifferentiation—a sequence that mirrors nephron progenitor expansion.^31^^,^^32^ At the molecular level, injured PTECs re-express fetal genes such as SRY-box transcription factor 9 (*Sox9*)—a transcription factor active during kidney development.^33^^,^^34^ At the pathway level, they reactivate RAR-dependent transcriptional networks that also operate in the embryonic kidney.^10^^,^^11^^,^^12^ Underpinning this reactivation is a dedicated compensatory mechanism: although injured PTECs transiently lose the RA-synthesizing enzyme ALDH1A2, neighboring interstitial fibroblasts acquire this capacity to ensure local RA availability.^10^^,^^11^ This RA reactivates RAR signaling in PTECs, driving a program of dedifferentiation, proliferation, and metabolic reprogramming that supports tubular repair^12^^,^^35^ (Figure 2). While the precise contribution of each RAR subtype to this repair program remains to be fully defined, RARα appears to play a primary homeostatic role in PTECs, with RARβ and RARγ also expressed and potentially contributing to the injury response.^8^^,^^11^^,^^36^

**Figure 2:**
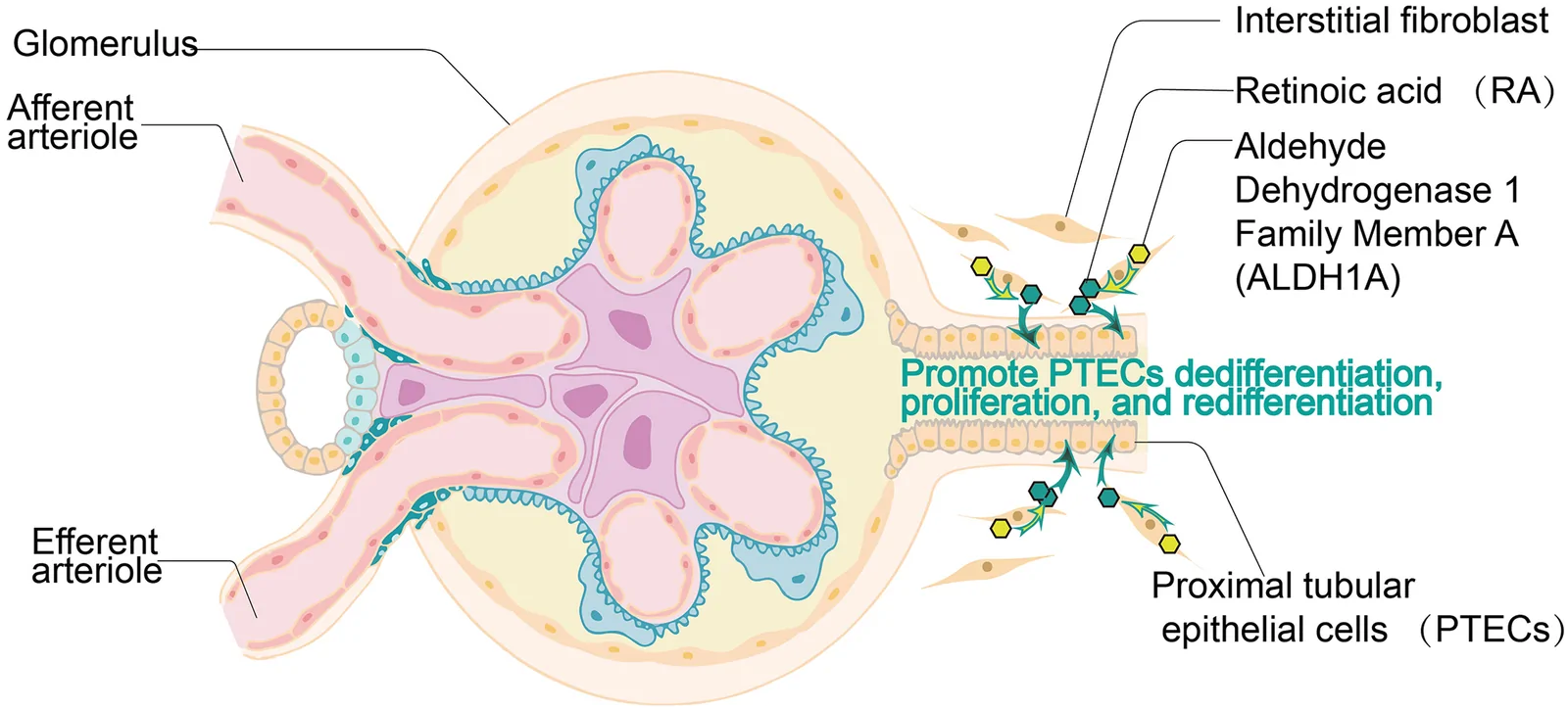
The role of RA in the repair of kidney injury Injured PTECs transiently lose the RA-synthesizing enzyme, ALDH1A2. Neighboring interstitial fibroblasts compensate by upregulating ALDH1A2 to ensure local RA availability. Fibroblast-derived RA reactivates RAR signaling in the injured PTECs and drives a repair program in PTECs, characterized by dedifferentiation, proliferation, and metabolic reprogramming, which collectively supports tubular epithelial regeneration. Abbreviations are as follows: RA, retinoic acid; PTEC, proximal tubular epithelial cell; ALDH1A2, aldehyde dehydrogenase 1 family member A2; RAR, retinoic acid receptor.

Despite this reactivation of developmental cues, the mammalian repair program often fails to progress beyond adaptive repair toward full regeneration. Unlike the robust regenerative response observed in axolotls—in which this intrinsic program proceeds to completion, culminating in *de novo* nephron formation^1^—the mammalian response is prone to derailment and rarely achieves new nephron formation, instead tending toward maladaptive repair. This limitation may stem from differences in signal dynamics. Embryonic RA gradients are robust and long-lasting.^37^^,^^38^^,^^39^ By contrast, post-injury RA activation is spatially restricted and short-lived.^40^ Moreover, in CKD, dominant inhibitory pathways—such as transforming growth factor β (TGF-β) and Wnt/β-catenin—actively suppress pro-regenerative RA signaling.^41^ Thus, while RA reactivation initiates the endogenous repair program, it falls short of steering it through to full completion along the regenerative continuum.

The central role of RA in nephrogenesis, coupled with the re-engagement of RA signaling after injury, raises the question of whether its developmental functions can be re-deployed in the adult kidney to guide tissue repair. This possibility is grounded in a broader paradigm in regenerative medicine, referred to as developmental bioinspiration—the strategy of instructing tissue repair by recapitulating spatial and temporal cues of embryonic development.^42^^,^^43^ In the kidney, this concept has been explicitly articulated as “developmental engineering the kidney”: leveraging principles of morphogenesis for renal regeneration.^44^^,^^45^ A critical step toward this goal is to understand the precise mode of RA action during nephrogenesis. While this remains incompletely defined, emerging evidence from model systems has begun to shed light on its spatiotemporal complexity. Insights from zebrafish have revealed that RA functions as a key spatiotemporal patterning signal during nephrogenesis: it regionalizes the renal progenitor field into proximal and distal domains, with the rostral domain relying on RA signaling and the caudal domain being repressed by elevated RA levels.^46^ Complementing this, studies in the developing rat kidney have dissected the metabolic basis of this spatiotemporal control. RA-synthesizing enzymes (ALDH1A1-3) and degrading enzymes (CYP26A1/B1) exhibit cell-type-specific and temporally dynamic expression patterns. For example, ALDH1A2 is restricted to the cortical stroma and later to developing glomeruli. In contrast, CYP26 enzymes are absent until late nephrogenesis, emerging only in S-shaped bodies and tubular anlagen.^47^ Together, these findings indicate that RA availability during nephrogenesis is under tight spatiotemporal control, defined by localized synthesis, degradation, and functional regionalization rather than a static, sustained signal.

## Strategies to harness RA for kidney regeneration

### Rationale for prioritizing RA over other candidates

Besides RA, several other molecules have shown preliminary regenerative potential in the kidney and can be broadly categorized into growth factors, endogenous regulatory systems, and small-molecule compounds.

Growth factors represent one of the most extensively studied categories. Hepatocyte growth factor (HGF) functions as a renotropic factor that supports renal regeneration by enhancing cell growth, preventing epithelial cell death, and promoting the regeneration and remodeling of renal tissue following injury or fibrosis.^48^ *In vivo* studies have demonstrated that proximal tubule-targeted HGF overexpression in transgenic mice stimulates the recovery of normal kidney function and the regeneration of proximal tubular epithelium after ischemic injury.^49^ Bone morphogenetic protein 7 (BMP-7), an inducer of nephrogenesis, functions as an endogenous renoprotective factor with anti-inflammatory, anti-fibrotic, and regenerative mechanisms. BMP-7 gene overexpression inhibits CKD progression and promotes tubular regeneration driven by proliferative *SOX9*-expressing tubular cells.^50^

Endogenous regulatory systems that govern kidney development and repair have emerged as promising therapeutic targets. The activin-follistatin system plays critical roles in renal organogenesis and regeneration following injury.^51^ Activin A inhibits tubular regeneration after renal ischemia. Follistatin, an endogenous antagonist of activin A, reverses this inhibitory effect and accelerates tubular regeneration.^52^

Small-molecule compounds targeting specific signaling pathways offer another avenue. Prolyl hydroxylase domain inhibitors (e.g., MK-8617) stabilize HIF by inhibiting prolyl hydroxylase, thereby creating a hypoxic-like state that facilitates renal tubular repair. This process is achieved by inducing the conversion of proximal tubular cells into *Sox9*-expressing progenitor cells.^34^

Among these diverse approaches, RA stands out for several unique advantages. First, as a small-molecule nuclear hormone, RA acts at the transcriptional level via RAR/RXR nuclear receptors, enabling it to influence a broad range of gene expression programs in a cell-type-dependent manner.^24^^,^^25^ This distinguishes it from growth factors (e.g., HGF and BMP-7), which bind surface receptors and primarily drive isolated downstream signaling cascades. Consequently, RA exerts pleiotropic effects across multiple cell types—epithelial (promoting dedifferentiation and proliferation^10^), immune (promoting M2 macrophage polarization^53^), and stromal (exerting context-dependent anti-fibrotic effects^8^)—coordinating a cohesive regenerative response that single-target agents cannot achieve. Furthermore, through its extensive downstream regulatory network, RA can modulate several of the aforementioned pro-regenerative candidate molecules, such as HGF^54^^,^^55^ and BMP-7,^56^ while simultaneously suppressing pro-fibrotic signals like TGF-β1. Second, recent proof-of-principle studies demonstrate that reactivating RA synthesis alone can unlock mammalian regeneration in non-regenerating adult tissues, suggesting RA signaling may function as a master regenerative switch.^15^ Third, as a small molecule, RA offers practical advantages over growth factors and other protein-based agents in terms of stability, scalability, and immunogenicity.

### Enhancing stem cell therapy via RA preconditioning

Stem cell therapy is one of the core technologies in regenerative medicine and has been extensively explored in preclinical models of kidney regeneration. Stem cell therapy for kidney regeneration aims to repair injured tissues and restore organ function through the transplantation of stem cells, a strategy with rapid clinical translation progress in this field.^57^^,^^58^ The general procedure involves isolating suitable stem cells, expanding and priming them *in vitro*, and delivering them via intravenous or intra-arterial infusion, relying on their homing capacity to reach the injured kidney.^59^^,^^60^ Currently, bone marrow-derived mesenchymal stem cells (MSCs) are the most commonly used source due to their easy accessibility, multilineage differentiation potential,^61^^,^^62^ and paracrine activities that modulate immune responses, suppress inflammation, and inhibit fibrosis.^62^^,^^63^^,^^64^ MSCs also possess inherent homing ability that enables targeted migration to injury sites.^65^ Clinical trials of MSC-based therapies have reported promising outcomes, including improved renal function, reduced injury markers, and partial histological repair. However, current stem cell therapies face inherent limitations, including low post-transplantation survival, limited homing efficiency, and unclear differentiation trajectories.^66^ To address these challenges, preconditioning MSCs with all-trans RA (ATRA) prior to transplantation has been explored. This ATRA preconditioning enhances cell viability and improves overall therapeutic efficacy (Figure 3). One proposed mechanism involves modulating the Wnt/β-catenin signaling pathway and reducing inflammatory cytokines, thereby alleviating oxidative stress and cell death in renal ischemia models.^67^ Another study demonstrated that ATRA pretreatment promotes the secretion of hyaluronic acid (HA), a key protective molecule that enhances cellular repair.^66^ In summary, ATRA pretreatment improves MSC viability and reparative capacity, offering a promising strategy to enhance stem cell-based kidney regeneration.

**Figure 3:**
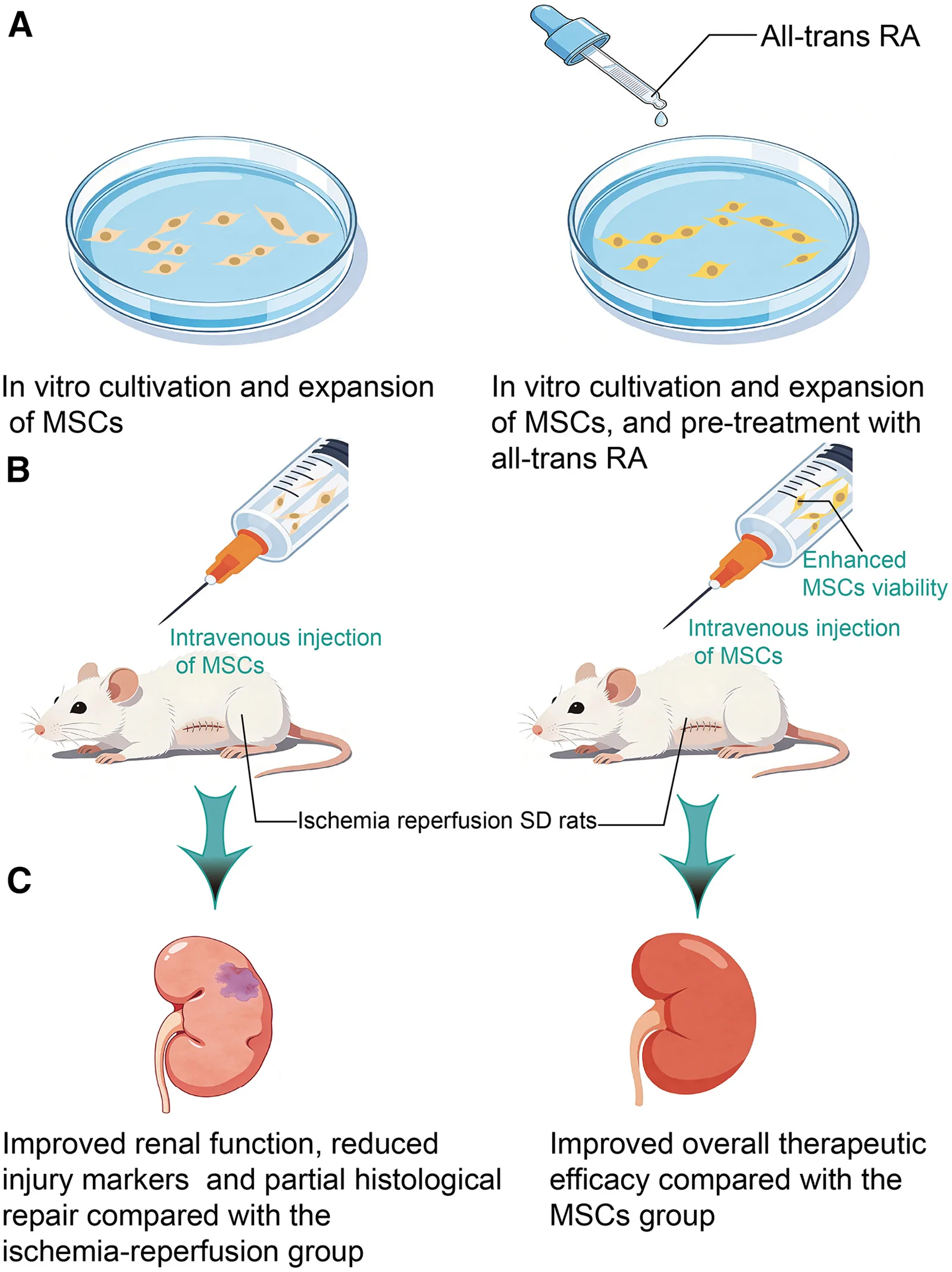
RA pretreatment can enhance the efficacy of stem cell therapy (A) Preconditioning: MSCs are treated with all-trans RA prior to transplantation. (B) Mechanism: all-trans RA pretreatment enhances MSC viability and modulates paracrine function. (C) Therapeutic outcome: the pretreated MSCs exhibit improved overall reparative capacity after transplantation, leading to enhanced renal repair. Abbreviations are as follows: MSCs, mesenchymal stem cells; RA, retinoic acid.

### Rationale for direct *in vivo* RA delivery to activate endogenous renal cells

While kidney organoids have demonstrated the essential role of RA in nephron patterning *in vitro*,^29^ assembling fully functional renal tissue outside the body remains challenging. This has prompted increasing interest in directly activating endogenous renal cells *in vivo*. A critical distinction must be made regarding the cellular players in stem cell-enhanced kidney repair: MSCs facilitate repair primarily through paracrine mechanisms (anti-inflammatory, anti-fibrotic etc.) rather than direct differentiation into new renal cells.^32^^,^^63^^,^^68^ In contrast, the principal executors of regeneration are endogenous renal cells. The most prominent are PTECs,^69^^,^^70^^,^^71^^,^^72^ which, after acute injury, dedifferentiate into a progenitor-like state (e.g., *Sox9*^*+*^), proliferate, and redifferentiate to repopulate the tubule—a process promoted by reactivated RA signaling.^34^^,^^71^ Beyond PTECs, regenerative potential resides in niches throughout the kidney.

#### Glomerular compartment

A specific subset of parietal epithelial cells (PECs) expressing CD133 and CD24 can migrate and differentiate into podocytes, contributing to repair of the filtration barrier.^2^^,^^70^^,^^73^ Notably, RA directly inhibits PEC proliferation and promotes their differentiation toward a podocyte fate.^74^^,^^75^ This RA-driven inhibition of PEC proliferation and induction of PEC-to-podocyte transdifferentiation attenuates crescent formation in crescentic glomerulonephritis.^74^ Conversely, this RA-dependent regenerative process is impaired by proteinuria, which sequesters RA and blocks the regenerative response.^76^

#### Interstitium

Renal interstitial progenitor-like cells (IPLCs) promote regeneration via epithelial-mesenchymal interactions. While direct evidence of RA action is limited, RA is routinely used as a differentiation cue in induced pluripotent stem cell (iPSC)-based induction protocols for IPLC lineages.^77^

#### Collecting duct system

Progenitor cells contribute to the network and its repair.^78^^,^^79^ As discussed in Section developmental blueprint: RA orchestrates nephrogenesis, RA signaling critically regulates branching morphogenesis of the collecting duct system. However, whether this developmental role extends to the regulation of collecting duct progenitor repair in the adult kidney remains to be determined.

#### Peri-ureteric niche

In *Drosophila*, quiescent renal stem cells (RSCs) are activated and migrate upon ureteric injury to remodel the tissue.^80^ However, to our knowledge, no studies have reported effects of RA on *Drosophila* RSCs.

These pivotal endogenous cells are scarce, difficult to isolate, and lose stemness *in vitro*, making them unsuitable for expansion and reinfusion as cell therapy.^65^ This context provides a powerful rationale for directly applying RA *in vivo*. RA’s capacity to enhance MSC efficacy *in vitro*, combined with its established role as a master regulator of endogenous progenitor patterning and differentiation during development, positions it as an ideal candidate to orchestrate regeneration *in situ* by recapitulating developmental signaling patterns. The goal is to prime renal tissue by: (1) promoting dedifferentiation of mature epithelial cells into a progenitor-like state; (2) establishing a pro-regenerative niche by suppressing inflammation and fibrosis; and (3) guiding the patterned differentiation of activated cells into functional structures.

## The dual nature of RA signaling in kidney disease: A determinant-based framework

RA has been tested in numerous kidney disease models. The outcomes, however, are strikingly divergent—ranging from robust protection to active pathology. This duality is not stochastic. It is governed by a set of definable parameters. We propose that three interconnected determinants—(1) disease context and inflammatory milieu, (2) target cell type, and (3) signaling intensity—collectively dictate whether RA signaling culminates in repair or damage. Understanding these determinants is essential for designing precision delivery strategies that harness RA’s benefits while suppressing its harmful effects.

### Determinant 1: Disease context and inflammatory milieu

The same RA signal can produce opposing outcomes depending on the underlying pathological environment. In ischemia-reperfusion injury, RA is protective. RA promotes macrophage polarization toward a reparative M2 phenotype, and inhibition of this signaling exacerbates tubular damage and fibrosis.^53^ Proximal tubule-specific deletion of RARα triggers mitochondrial dysfunction, inflammation, and progressive fibrosis, phenocopying human CKD.^8^ In diabetic nephropathy, RA attenuates fibrosis by suppressing the TGF-β1/Smad3 pathway.^81^ In crescentic glomerulonephritis, RA activates podocyte RARα. This protects against injury and promotes regeneration by inducing transdifferentiation of PECs into podocytes.^74^

In septic acute kidney injury, the milieu is dominated by systemic inflammation. In this context, widespread RAR reactivation in proximal tubules becomes pathological. Its inhibition alleviates injury.^12^ This dichotomy suggests that the inflammatory microenvironment acts as a critical rheostat that tunes RA signaling output. However, the precise molecular cues that redirect RA from protective to detrimental programs remain to be fully elucidated.

### Determinant 2: Target cell type

The same RA signal can elicit distinct—and sometimes opposing—functional outcomes depending on the responding cell type. This is exemplified by comparing PTECs and interstitial fibroblasts. In PTECs, RARα activation maintains metabolic homeostasis and prevents fibrosis.^8^ By contrast, in interstitial fibroblasts, RA released from injured tubules induces RA induced 14 (RAI14). RAI14 stabilizes TRIO and F-actin-binding protein (TRIOBP), enhances cytoskeletal tension, and promotes nuclear translocation of Yes-associated protein (YAP). This cascade drives fibroblast-to-myofibroblast transition and fibrosis. Notably, inhibiting RAI14 blunts fibrosis without compromising baseline kidney function.^82^ Thus, RA is protective in PTECs but pathogenic in fibroblasts.

### Determinant 3: Signaling intensity

The dose-response relationship of RA is bell-shaped rather than monotonic. This is exemplified by studies in podocytes. Physiological or low-dose RA activates protective RARα/cAMP/PKA/KLF15 signaling. In contrast, high-dose RA upregulates RA receptor responder 1 (RARRES1).^83^ RARRES1 is cleaved by matrix metalloproteinase 23 (MMP23) into a soluble form (soluble RARRES1 [sRARRES1]). sRARRES1 then triggers p53-mediated apoptosis in podocytes, accelerating glomerulosclerosis. Notably, inhibiting MMP23 blocks this cleavage and prevents sRARRES1-induced apoptosis,^84^^,^^85^ while inflammatory cues such as tumor necrosis factor α (TNF-α) cooperate with RA to further induce RARRES1 expression.^83^^,^^86^

### Bridging biology to delivery design

The three determinants outlined above define a preliminary design brief for RA delivery systems. These considerations translate into four engineering goals: (1) co-modulating the microenvironment (e.g., suppressing inflammatory cues such as TNF-α), (2) cell-selective targeting to avoid activating interstitial fibroblasts and fibrosis, (3) precise dose control, and (4) intercepting pathological effector pathways such as MMP23 and RAI14. However, the exact parameters for achieving these goals—such as optimal dose thresholds, signal duration, and interactions between determinants—remain incompletely defined in the current literature. Nevertheless, these principles establish part of the rationale for the advanced biomaterial platforms discussed in engineering delivery platforms: Nanocarriers and scaffolds.

## Pharmacological and toxicity barriers of RA

The clinical application of RA is severely hampered by several intrinsic pharmacological limitations. First, RA exhibits poor aqueous solubility, which impedes its effective absorption and systemic distribution.^19^ Second, it is highly photosensitive, leading to instability and complicating both storage and clinical use.^20^ Third, RA has a short half-life; it undergoes rapid hepatic metabolism (primarily by CYP450 enzymes) following administration, resulting in a swift decline in plasma concentration—a phenomenon known as the first-pass effect. Oral RA formulations require higher doses to compensate for this effect, which escalates the risk of adverse events.^21^ Finally, RA lacks tissue specificity. Systemic administration leads to non-specific actions across multiple organs, triggering off-target side effects.^87^ Consequently, the therapeutic utility of free RA is limited by its low efficacy and high toxicity profile.

These pharmacokinetic shortcomings directly contribute to RA’s dose-dependent systemic toxicity. The liver, as the primary site of vitamin A storage and metabolism, is highly susceptible; excessive exogenous RA can disrupt hepatic vitamin A homeostasis, leading to elevated serum transaminases, hepatic steatosis, or injury.^16^ Beyond the liver, RA profoundly affects epithelial tissues: while promoting keratinocyte differentiation, it impairs the skin barrier, causing irritation, dryness, desquamation, and ocular inflammation.^17^ RA treatment can also induce mucositis.^88^ The most severe toxicity is its potent teratogenicity. Systemic administration during pregnancy can cause severe congenital malformations, particularly in craniofacial, central nervous, and cardiac structures.^89^ Furthermore, excessive RA signaling may disrupt bone homeostasis by accelerating osteoblast differentiation or impairing chondrocyte function.^16^ Additionally, RA therapy is associated with an increased risk of depression and suicidal ideation; intraperitoneal RA injection in mice induces depression-like behaviors^90^^,^^91^ (Table 1). In summary, the convergence of poor pharmacokinetics and multi-organ toxicity creates a narrow therapeutic window that renders conventional systemic RA administration ineffective and potentially harmful.

**Table 1:** Overview of systemic toxicity of retinoic acid

Toxicity category	Clinical manifestations	Mechanisms	Clinical notes/monitoring
Mucocutaneous	skin dryness, pruritus, cheilitis, xerostomia, epistaxis, and conjunctivitis	inhibition of sebaceous gland and mucosal epithelial cell function	most common; typically dose-dependent
Central nervous system	headache, pseudotumor cerebri, dizziness, depression, and fatigue	not fully elucidated	pseudotumor cerebri may be associated with concomitant tetracycline use; monitor for headache and papilledema
Musculoskeletal	bone pain, arthralgia, myalgia, premature epiphyseal closure (in children), and hypercalcemia	accelerated osteoblast differentiation; impaired chondrocyte function	long-term, high-dose therapy requires monitoring of bone density and growth, especially in children
Hepatotoxicity and metabolic	elevated transaminases and hypertriglyceridemia	disruption of hepatic vitamin A homeostasis; altered lipid metabolism	regular monitoring of liver function and lipids is crucial; hyperlipidemia may increase pancreatitis risk
Gastrointestinal	nausea, vomiting, and abdominal pain	mucosal irritation	common but usually mild to moderate
Teratogenicity	severe congenital malformations (craniofacial, cardiac, and central nervous system)	disruption of embryonic development via RAR-mediated gene regulation	most serious toxicity; strict contraception mandatory for women of childbearing potential during and after treatment

Abbreviations: RA, retinoic acid; RAR, retinoic acid receptor; CKD, chronic kidney disease; ESRD, end-stage renal disease.

## Engineering delivery platforms: Nanocarriers and scaffolds

The design of delivery platforms for RA must be guided by the biological constraints established in the preceding sections—RA’s narrow therapeutic window, its cell-type-specific and context-dependent duality, the distinction between embryonic and post-injury signaling dynamics, and its systemic toxicity. These constraints collectively define a set of engineering requirements. An effective delivery system must achieve: (1) local confinement to bypass rapid hepatic metabolism and prevent systemic toxicity; (2) cell-type selectivity to activate regenerative programs in target cells (e.g., PTECs) while avoiding pathogenic activation in others (e.g., interstitial fibroblasts); (3) spatiotemporal control that recapitulates the dynamic, spatiotemporally restricted pattern of developmental RA signaling rather than sustained exposure; (4) pathway-specific modulation to intercept maladaptive downstream effectors (e.g., RAI14 and sRARRES1) without broadly suppressing RA signaling; and (5) microenvironmental remodeling to counteract the inflammatory and fibrotic signals that derail endogenous repair, creating a permissive niche that supports RA-driven regeneration. These design principles are summarized in Table 2.

**Table 2:** Engineering design principles for RA delivery systems aligned with biological constraints

Design principle	Core strategy and rationale	Expected outcome
Local confinement	using renal-targeted nanocarriers to concentrate RA within the kidney, thereby bypassing rapid hepatic metabolism and reducing systemic exposure to off-target organs	minimizes systemic toxicity and first-pass metabolism, improving the therapeutic index of RA
Cell-type selectivity	decorating nanocarriers with ligands that bind to receptors on target cells (e.g., PTECs) while avoiding pathogenic cell types (e.g., interstitial fibroblasts), to deliver RA precisely where it is needed	activates regenerative programs in target cells and avoids pathogenic activation in off-target cells, addressing RA’s cell-specific duality
Spatiotemporal control	engineering scaffold-based systems to release RA in a spatially patterned and temporally controlled manner, recapitulating the dynamic, spatiotemporally restricted patterns of developmental RA signaling	provides localized, morphogen-like signaling that mirrors native RA dynamics, overcoming the transient and spatially limited nature of post-injury RA activation
Pathway-specific modulation	co-delivering specific inhibitors (e.g., against MMP23 or RAI14) alongside RA to intercept pathological downstream effectors without broadly suppressing RA signaling	selectively disarms RA’s fibrotic/apoptotic arms (sRARRES1, RAI14) while preserving its beneficial regenerative signals
Microenvironmental remodeling	co-delivering RA with synergistic effectors (decellularized kidney ECM, NO-releasing components, MSC-derived EVs, and antioxidant molecules) to counteract inflammation and fibrosis	creates a permissive niche that enhances progenitor cell recruitment, survival, and differentiation, complementing the spatiotemporal guidance provided by RA
Overarching concept: developmental bioinspiration	reconstructing the complex, dynamic, and instructive signaling landscape of nephrogenesis within the injured adult kidney through integrated biomaterial design	guides the injured adult kidney along the regenerative continuum by recapitulating ontogenic programs

Abbreviations: PTECs, proximal tubular epithelial cells; MMP23, matrix metalloproteinase 23; RAI14, retinoic acid-induced 14; sRARRES1, soluble retinoic acid receptor responder 1; NO, nitric oxide; MSC, mesenchymal stem cell; ECM, extracellular matrix; EVs, extracellular vesicles.

Further, we evaluate two complementary platform classes—nanocarriers and scaffolds—against these design criteria.

### Nanocarriers for kidney-targeted and pulsatile delivery

Nanocarriers are particularly well-suited to address two of the aforementioned biological constraints: local confinement and cell-type selectivity. Nanocarriers can overcome RA’s poor solubility, rapid metabolism, and off-target toxicity. They protect RA, prolong its circulation, and deliver it specifically to the injured kidney. Indeed, the feasibility of RA-loaded nanocarriers for kidney targeting has been demonstrated: oral ATRA-loaded chitosan/tripolyphosphate lipid hybrid nanoparticles showed significantly enhanced therapeutic efficacy in a rat model of diabetic nephropathy compared with free ATRA.^92^

#### Passive targeting to the kidney

The unique physiological architecture of the kidney presents distinct advantages for nanotherapeutic targeting. The organ’s high perfusion rate (receiving ∼25% of cardiac output) and the specialized structure of the glomerulus facilitate the passive accumulation of small nanoparticles. Specifically, the glomerular filtration barrier, a triple-layered sieve composed of the fenestrated endothelium, the glomerular basement membrane (GBM), and the podocyte slit diaphragm, provides the structural basis for this size-selective passive targeting. The innermost endothelial layer contains pores (fenestrations) ranging from 70–100 nm in diameter. While the GBM has a more compact meshwork (approximately 4–8 nm), its gel-like, deformable nature allows for the passage of nanoparticles under pressure. The outermost podocyte layer, with slit diaphragms of about 25 nm, constitutes the decisive size-selective barrier. Owing to this unique structure, nanoparticles sized 70–100 nm can traverse the endothelial fenestrations but are subsequently retained in the mesangial region due to the restriction imposed by the podocyte slit diaphragms, thereby achieving passive targeting to the mesangium (Figure 4).^93^^,^^94^ While this passive strategy enhances the renal targeting efficiency of nanoparticles and reduces off-target effects, it does not achieve cell-type selectivity. Active targeting thus represents a superior strategy for directing RA to the specific cell types where it is needed.

**Figure 4:**
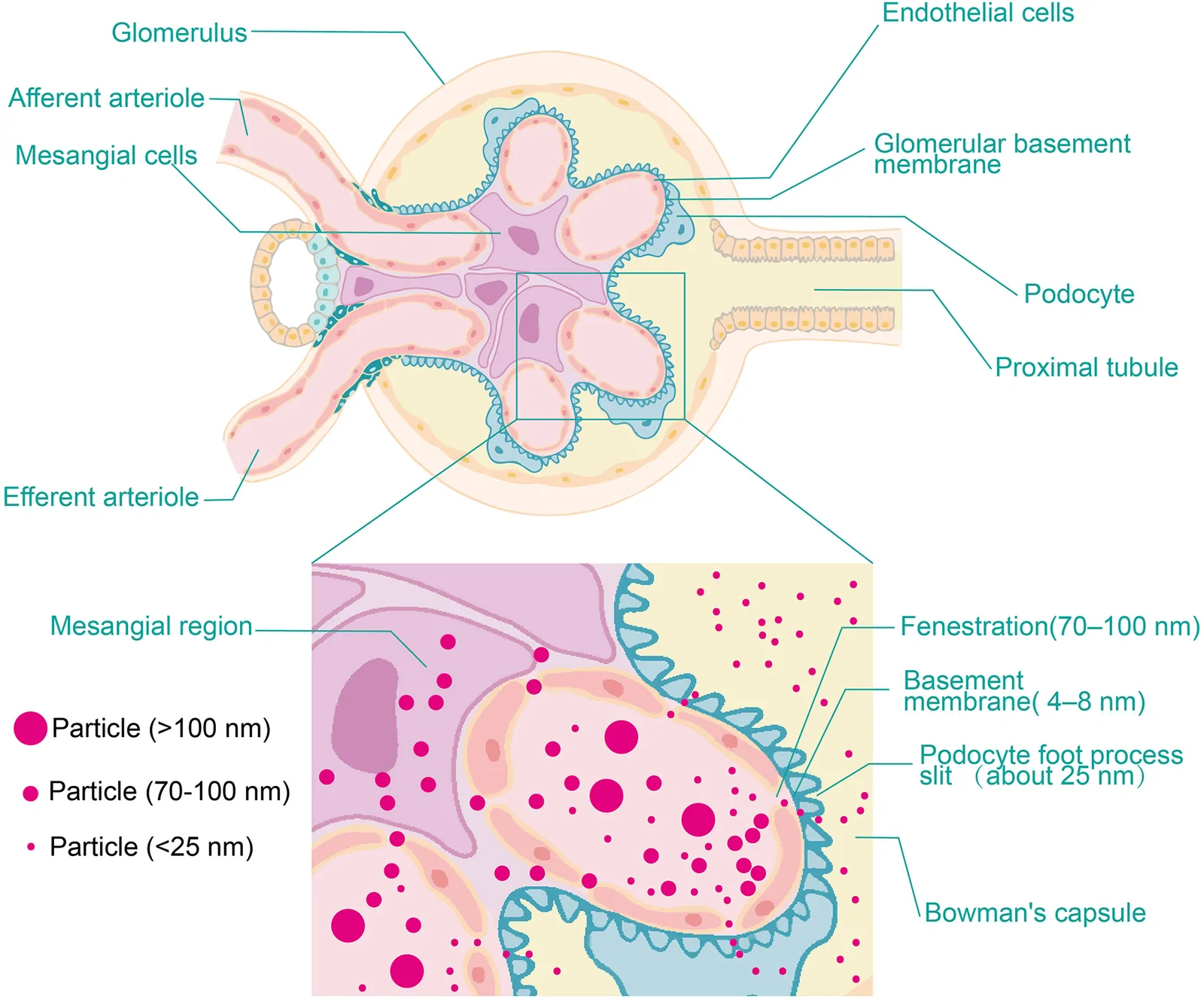
Mechanism of passive nanoparticle targeting to the renal mesangium Structural basis: the glomerular filtration barrier consists of a fenestrated endothelium (70–100 nm pores), the glomerular basement membrane (GBM), a deformable gel with pores of ∼4–8 nm, and podocyte slit diaphragms (∼25 nm gaps). Targeting mechanism: systemically administered nanoparticles sized 70–100 nm traverse the endothelial fenestrations and, under physiological pressure, pass through the deformable GBM. They are ultimately retained in the mesangium as their size exceeds the filtration limit of the slit diaphragms. This schematic illustrates the size-selective principle of passive targeting. However, renal accumulation and localization are also influenced by other physicochemical properties of nanoparticles (e.g., surface charge, shape, and surface chemistry) as well as disease-related factors that can alter glomerular permeability and hemodynamics. Abbreviations are as follows: GBM, glomerular basement membrane.

#### Active renal targeting of nanocarriers

To achieve cell-type selectivity—and to concentrate RA precisely at the cells where it is beneficial (e.g., PTECs) while avoiding those where it is pathogenic (e.g., interstitial fibroblasts)—nanocarriers can be decorated with ligands that bind to receptors on specific renal cell types, thereby enhancing localized uptake.

PTECs are a primary injury site in kidney disease and express several targetable receptors. For example, the megalin-cubilin complex can be engaged by glucosamine or chitosan.^95^ Folate receptor-α is also highly expressed; folate-decorated pluronic nanoparticles achieved a 5-fold higher kidney drug concentration than in the liver.^96^ More precise targeting can exploit injury-upregulated receptors: a biomimetic high-density lipoprotein (bHDL) nanocarrier targeting kidney injury molecule-1 (KIM1) enabled efficient drug delivery to injured tubules.^97^ Peptide-based targeting offers additional versatility, and recent AI-driven approaches have further accelerated the discovery of high-affinity targeting ligands.^98^^,^^99^^,^^100^^,^^101^

### Multifunctional scaffolds: Engineering a pro-regenerative niche

Beyond nanocarriers, multifunctional scaffolds are uniquely suited to address the remaining three design requirements, spatiotemporal control, pathway-specific modulation, and microenvironmental remodeling. They could be designed to mimic the functions of the natural extracellular matrix (ECM), providing both biochemical signals and physical support to guide complex tissue regeneration.

A proof-of-concept from CKD: a landmark study in a 5/6 nephrectomy model used a poly(lactic-co-glycolic acid) (PLGA)-based composite scaffold loaded with multiple components: Mg(OH)_2_ to neutralize acidic degradation products, decellularized kidney ECM for tissue-specific cues, ZnO-ALA nanoparticles to release nitric oxide (NO) for blood vessel growth, and melatonin-primed MSC-derived extracellular vesicles (EVs) for anti-inflammatory and pro-survival signals. Remarkably, this acellular scaffold improved kidney function, reduced fibrosis, and even promoted glomerular regeneration—showing that a well-designed environment can trigger the body’s own repair mechanisms without cell transplantation.^102^

Toward an RA-integrated scaffold: building on this work, we envision a next-generation scaffold that could incorporate RA to actively steer regeneration. The design could follow three principles.

• Spatiotemporal control: the scaffold could release RA in a spatiotemporally patterned way, creating morphogen gradients like those in development. This could be achieved using engineered microspheres that provide programmed, long-lasting RA release.^103^^,^^104^ This design principle directly addresses the signaling dynamics mismatch between robust, long-lasting embryonic gradients and short-lived, spatially restricted post-injury activation discussed in Section post-injury reactivation: Developmental programs replayed.
• Targeted pathomechanism interception: a promising safety-by-design feature would be the local co-delivery of RA with inhibitors targeting its pathological downstream effectors, including MMP23 (responsible for sRARRES1 generation) or RAI14. This approach directly responds to the maladaptive effectors identified in Section the dual nature of RA signaling in kidney disease: A determinant-based framework—such as sRARRES1-mediated podocyte apoptosis and RAI14-driven fibroblast activation—offering a strategy to intercept RA’s pathogenic outputs without broadly suppressing its regenerative signals.
• Microenvironmental remodeling via synergistic codelivery: RA could be co-delivered with other effectors that have already shown promise in kidney scaffold studies. For example, decellularized kidney ECM can provide tissue-specific guidance for authentic cell recruitment and spatial organization^102^^,^^105^; NO-releasing components (e.g., ZnO-ALA nanoparticles) can promote vascularization alongside RA-driven tubulogenesis^102^^,^^106^; and MSC-derived EVs can modulate immune responses and suppress fibrosis.^107^^,^^108^ Looking further ahead, one could envision incorporating additional antioxidant molecules (e.g., N-acetylcysteine) to further “clean up” the diseased adult environment, making it more like the permissive state of an embryo—removing barriers to regeneration.^109^^,^^110^ Collectively, these synergistic effectors address the hostile microenvironment (Section determinant 1: Disease context and inflammatory milieu) that derails endogenous repair, creating a permissive niche that complements the spatiotemporal guidance provided by RA.

## Developmental bioinspiration: Challenges and roadmap

The concept of developmental bioinspiration—actively recapitulating the dynamic, multi-signal, and spatiotemporally patterned processes of embryonic development for tissue repair—has gained increasing recognition in tissue engineering and regenerative medicine over the past decade. The term builds on the broader framework of developmental engineering, which proposes using intermediate developmental tissue forms and autonomously progressing developmental modules as prime targets for constructing clinically efficacious bioartificial tissues.^111^^,^^112^ This thinking marked a paradigm shift away from simply trying to replicate adult tissue features, toward leveraging nature’s own developmental blueprints.^42^ The approach has since been applied across multiple organ systems, including bone, cartilage, liver and kidney.^113^^,^^114^^,^^115^ In the renal field, pioneering work by Little and colleagues^116^^,^^117^ has championed the strategy of “returning to kidney development to deliver synthetic kidneys”, explicitly using developmental rules to guide the *in vitro* generation of kidney tissue from pluripotent stem cells. Building on this strategy, they have successfully generated iPSC-derived kidney organoids that contain segmented nephrons, collecting ducts, and stromal compartments. They have further developed proximal tubule-enhanced protocols to improve organoid maturity and function, and applied extrusion-based bioprinting to enable reproducible, high-throughput organoid production. However, significant challenges remain, including achieving functional maturity, organotypic vascularization, and scalable production. Despite these hurdles, this body of work represents a pivotal proof of concept that developmental principles can be harnessed to reconstruct kidney tissue *in vitro*.

However, translating this roadmap into clinical reality faces substantial gaps. First, the optimal dose, timing, and spatial pattern of RA delivery remain unknown. Native RA signaling is tightly restricted in dose, space, and time as discussed in Section post-injury reactivation: Developmental programs replayed. In zebrafish embryos lacking endogenous RA, a brief 5-min incubation with 1 nM ATRA is sufficient to rescue hindbrain development,^118^ suggesting that continuous exposure may not be necessary. Whether pulsatile, ramp-up, intermittent, or other temporally patterned profiles—and at what concentration—are optimal for kidney regeneration remains completely unknown. Moreover, engineering such precisely controlled profiles is technically challenging, and their application to renal repair remains to be systematically evaluated. Second, the hostile CKD/end-stage renal disease (ESRD) microenvironment suppresses endogenous RA signaling and may blunt the response even to optimally delivered RA signaling. Whether co-delivery of anti-inflammatory or anti-fibrotic agents can overcome this remains to be tested. Third, manufacturing complexity, batch variability, long-term safety, and immunogenicity pose formidable translational hurdles. Among these, the potential risks of nanoparticle retention in the kidney—including oxidative stress, inflammation, fibrosis, and cytotoxicity—warrant particular attention.^119^ These risks are largely dependent on nanoparticle physicochemical properties and can be mitigated through rational design. Key strategies include engineering nanoparticle size below the renal clearance threshold (∼6 nm) for rapid clearance,^120^ employing biodegradable materials to avoid long-term accumulation, and optimizing surface properties such as charge and **polyethylene glycolation** (PEGylation) to control biodistribution and reduce non-specific retention.^87^ Finally, demonstrating true regeneration (*de novo* nephron formation) will demand more than functional improvement or fibrosis reduction. Acknowledging these limitations does not diminish the promise of developmental bioinspiration; rather, it clarifies the critical experiments needed to turn an inspiring idea into a clinical solution.

## Conclusions and future perspectives

The therapeutic promise of RA for kidney regeneration is hindered by its context-dependent duality, systemic toxicity, and inherent pharmacological limitations. This review posits that overcoming these barriers requires precise spatiotemporal control over RA delivery, achievable through advanced biomaterial strategies.

We have consolidated evidence that RA possesses intrinsic regenerative potential in the kidney, yet its clinical translation has been blocked not by lack of efficacy but by poor pharmacokinetics and off-target toxicity. Among various regenerative candidates, RA stands out for its ability to activate intrinsic developmental programs and coordinate multi-cell-type responses—advantages that, however, have been thwarted by its pharmacological limitations. The context-dependent duality of RA—governed by inflammatory milieu, target cell type, and signaling intensity—demands delivery platforms that can achieve cell-selective, dose-controlled modulation. Renal-targeted nanocarriers can deliver RA locally to injured tubules, while multifunctional scaffolds offer a more ambitious strategy to fill structural defects and engineer a pro-regenerative niche. Together, these platforms embody a developmental bioinspiration paradigm that aims to recapitulate the native spatiotemporal dynamics of RA signaling, with the goal of steering the adult kidney away from maladaptive fibrosis and along the regenerative continuum toward structural and functional restoration.

This framework shifts the paradigm from passive drug administration to active, morphogen-guided tissue reconstruction. By providing a testable roadmap—including design principles and key unanswered questions—this review aims to stimulate the critical experiments needed to transform RA from a once-toxic molecule into a precision regenerative tool for kidney disease.

## Acknowledgments

This work was supported by the 10.13039/501100001809National Natural Science Foundation of China (grant nos. 81960679 and 82470716) and the Young Science and Technology Talent Training Program of Guangdong Provincial Science and Technology Association (grant no. SKXRC2025358).

## Declaration of interests

The authors declare no competing interests.

## References

[1] Chen L., Li J., Ou Y., Kang M., Deng J., Wang Y., Liang S., Hong X., Gong S., Fei J.-F. (2023). The axolotl kidney: a novel model to study kidney regeneration. Kidney Int..

[2] Romagnani P., Lasagni L., Remuzzi G. (2013). Renal progenitors: an evolutionary conserved strategy for kidney regeneration. Nat. Rev. Nephrol..

[3] Ferenbach D.A., Bonventre J.V. (2015). Mechanisms of maladaptive repair after AKI leading to accelerated kidney ageing and CKD. Nat. Rev. Nephrol..

[4] Lee K., Gusella G.L., He J.C. (2021). Epithelial proliferation and cell cycle dysregulation in kidney injury and disease. Kidney Int..

[5] Ledru N., Wilson P.C., Muto Y., Yoshimura Y., Wu H., Li D., Asthana A., Tullius S.G., Waikar S.S., Orlando G., Humphreys B.D. (2024). Predicting proximal tubule failed repair drivers through regularized regression analysis of single cell multiomic sequencing. Nat. Commun..

[6] Niederreither K., Dollé P. (2008). Retinoic acid in development: towards an integrated view. Nat. Rev. Genet..

[7] Ghyselinck N.B., Duester G. (2019). Retinoic acid signaling pathways. Development.

[8] DiKun K.M., Tang X.H., Fu L., Choi M.E., Lu C., Gudas L.J. (2024). Retinoic acid receptor α activity in proximal tubules prevents kidney injury and fibrosis. Proc. Natl. Acad. Sci. USA.

[9] Schmidt P., Leman E., Lagadec R., Schubert M., Mazan S., Reshef R. (2022). Evolutionary Transition in the Regulation of Vertebrate Pronephros Development: A New Role for Retinoic Acid. Cells.

[10] DiKun K.M., Gudas L.J. (2023). Vitamin A and retinoid signaling in the kidneys. Pharmacol. Ther..

[11] Nakamura J., Sato Y., Kitai Y., Wajima S., Yamamoto S., Oguchi A., Yamada R., Kaneko K., Kondo M., Uchino E. (2019). Myofibroblasts acquire retinoic acid-producing ability during fibroblast-to-myofibroblast transition following kidney injury. Kidney Int..

[12] Yang M., Lopez L.N., Brewer M., Delgado R., Menshikh A., Clouthier K., Zhu Y., Vanichapol T., Yang H., Harris R.C. (2023). Inhibition of retinoic acid signaling in proximal tubular epithelial cells protects against acute kidney injury. JCI Insight.

[13] Viragova S., Li D., Klein O.D. (2024). Activation of fetal-like molecular programs during regeneration in the intestine and beyond. Cell Stem Cell.

[14] Fazilaty H., Basler K. (2023). Reactivation of embryonic genetic programs in tissue regeneration and disease. Nat. Genet..

[15] Lin W., Jia X., Shi X., He Q., Zhang P., Zhang X., Zhang L., Wu M., Ren T., Liu Y. (2025). Reactivation of mammalian regeneration by turning on an evolutionarily disabled genetic switch. Science.

[16] Gudas L.J. (2022). Synthetic Retinoids Beyond Cancer Therapy. Annu. Rev. Pharmacol. Toxicol..

[17] Dai X., Jin J., Jia Y., Yang K., Han J., Zhang Z., Ding X., Yao C., Sun T., Zhu C., Liu H. (2022). A non-retinol retinoic acid receptor-γ (RAR-γ/NR1B3) selective agonist, tectorigenin, can effectively inhibit the ultraviolet A-induced skin damage. Br. J. Pharmacol..

[18] Xie X., Li C., Yu J., Chang S., Cheng X., Wang F., Bao Y., Zhang T., Wang S. (2023). MTHFD1 is critical for the negative regulation of retinoic acid receptor signalling in anencephaly. Brain.

[19] Xie B., Liu Y., Li X., Yang P., He W. (2024). Solubilization techniques used for poorly water-soluble drugs. Acta Pharm. Sin. B.

[20] Elfakhri K.H., Niu M., Ghosh P., Ramezanli T., Raney S.G., Ahmed S., Willett D.R., Yilmaz H., Ashraf M., Zidan A.S. (2022). Physicochemical and structural evaluation of microparticles in tretinoin topical gels. Int. J. Pharm..

[21] Gege C. (2021). Retinoic acid-related orphan receptor gamma t (RORγt) inverse agonists/antagonists for the treatment of inflammatory diseases - where are we presently?. Expert Opin. Drug Discov..

[22] Wei Q., Dong Z. (2020). The yin and yang of retinoic acid signaling in kidney diseases. J. Clin. Investig..

[23] Costantini F., Kopan R. (2010). Patterning a complex organ: branching morphogenesis and nephron segmentation in kidney development. Dev. Cell.

[24] Fusco A.N., Oxburgh L., Carroll T.J. (2025). The kidney stroma in development and disease. Nat. Rev. Nephrol..

[25] Bohnenpoll T., Weiss A.C., Labuhn M., Lüdtke T.H., Trowe M.O., Kispert A. (2017). Retinoic acid signaling maintains epithelial and mesenchymal progenitors in the developing mouse ureter. Sci. Rep..

[26] Weiss A.C., Rivera-Reyes R., Englert C., Kispert A. (2020). Expansion of the renal capsular stroma, ureteric bud branching defects and cryptorchidism in mice with Wilms tumor 1 gene deletion in the stromal compartment of the developing kidney. J. Pathol..

[27] Weiss A.C., Bohnenpoll T., Kurz J., Blank P., Airik R., Lüdtke T.H., Kleppa M.J., Deuper L., Kaiser M., Mamo T.M. (2019). Delayed onset of smooth muscle cell differentiation leads to hydroureter formation in mice with conditional loss of the zinc finger transcription factor gene Gata2 in the ureteric mesenchyme. J. Pathol..

[28] Mendelsohn C., Batourina E., Fung S., Gilbert T., Dodd J. (1999). Stromal cells mediate retinoid-dependent functions essential for renal development. Development.

[29] Huang B., Medina P., He J., Zeng Z., Kim S., Romo J., Koppitch K., Zhang C.C., Gyarmati G., Park Y. (2025). Spatially patterned kidney assembloids recapitulate progenitor self-assembly and enable high-fidelity in vivo disease modeling. Cell Stem Cell.

[30] Wilson S.B., Howden S.E., Vanslambrouck J.M., Dorison A., Alquicira-Hernandez J., Powell J.E., Little M.H. (2022). DevKidCC allows for robust classification and direct comparisons of kidney organoid datasets. Genome Med..

[31] Zhang D., Jiang H., Yang X., Zheng S., Li Y., Liu S., Xu X. (2024). Traditional Chinese Medicine and renal regeneration: experimental evidence and future perspectives. Chin. Med..

[32] Nie H., Zhao Z., Zhou D., Li D., Wang Y., Ma Y., Liu X., Zuo W. (2023). Activated SOX9+ renal epithelial cells promote kidney repair through secreting factors. Cell Prolif..

[33] Kim J.Y., Silvaroli J.A., Martinez G.V., Bisunke B., Luna Ramirez A.V., Jayne L.A., Feng M.J.H.H., Girotra B., Acosta Martinez S.M., Vermillion C.R. (2023). Zinc finger protein 24-dependent transcription factor SOX9 up-regulation protects tubular epithelial cells during acute kidney injury. Kidney Int..

[34] Li J., Chen L.T., Wang Y.L., Kang M.X., Liang S.T., Hong X.Z., Hou F.F., Zhang F.J. (2025). Inhibition of HIF-prolyl hydroxylase promotes renal tubule regeneration via the reprogramming of renal proximal tubular cells. Acta Pharmacol. Sin..

[35] Brilli Skvarca L., Han H.I., Espiritu E.B., Missinato M.A., Rochon E.R., McDaniels M.D., Bais A.S., Roman B.L., Waxman J.S., Watkins S.C. (2019). Enhancing regeneration after acute kidney injury by promoting cellular dedifferentiation in zebrafish. Dis. Model. Mech..

[36] Yago-Ibáñez J., García-Pastor C., Lucio-Cazaña F.J., Fernández-Martínez A.B. (2020). Retinoic acid receptor-beta prevents cisplatin-induced proximal tubular cell death. Biochim. Biophys. Acta. Mol. Basis Dis..

[37] Yamanaka Y., Hamidi S., Yoshioka-Kobayashi K., Munira S., Sunadome K., Zhang Y., Kurokawa Y., Ericsson R., Mieda A., Thompson J.L. (2023). Reconstituting human somitogenesis in vitro. Nature.

[38] Isla-Magrané H., Zufiaurre-Seijo M., García-Arumí J., Duarri A. (2022). All-trans retinoic acid modulates pigmentation, neuroretinal maturation, and corneal transparency in human multiocular organoids. Stem Cell Res. Ther..

[39] Schnirman R.E., Kuo S.J., Kelly R.C., Yamaguchi T.P. (2023). The role of Wnt signaling in the development of the epiblast and axial progenitors. Curr. Top. Dev. Biol..

[40] Luo W., Xu Y., Liu R., Liao Y., Wang S., Zhang H., Li X., Wang H. (2022). Retinoic acid and RARγ maintain satellite cell quiescence through regulation of translation initiation. Cell Death Dis..

[41] Somanader D.V.N., Zhao P., Widdop R.E., Samuel C.S. (2024). The involvement of the Wnt/β-catenin signaling cascade in fibrosis progression and its therapeutic targeting by relaxin. Biochem. Pharmacol..

[42] Chakraborty J., Chawla S., Ghosh S. (2022). Developmental biology-inspired tissue engineering by combining organoids and 3D bioprinting. Curr. Opin. Biotechnol..

[43] Chakraborty J., Ghosh S. (2025). Developmental Biology-Based 3D Bioprinting: An Off-the-Shelf Strategy to Induce Tissue Regeneration. ACS Biomater. Sci. Eng..

[44] Warrner E., Huang A.Z., Hughes A.J. (2026). Developmentally inspired synthetic kidney engineering. Nat. Biotechnol..

[45] Basu J., Ludlow J.W. (2012). Developmental engineering the kidney: leveraging principles of morphogenesis for renal regeneration. Birth Defects Res. C Embryo Today..

[46] Wingert R.A., Davidson A.J. (2011). Zebrafish nephrogenesis involves dynamic spatiotemporal expression changes in renal progenitors and essential signals from retinoic acid and irx3b. Dev. Dyn..

[47] Marlier A., Gilbert T. (2004). Expression of retinoic acid-synthesizing and -metabolizing enzymes during nephrogenesis in the rat. Gene Expr. Patterns.

[48] Mao S., Zhang J. (2016). The emerging role of hepatocyte growth factor in renal diseases. J. Recept. Signal Transduct. Res..

[49] Fiaschi-Taesch N.M., Santos S., Reddy V., Van Why S.K., Philbrick W.F., Ortega A., Esbrit P., Orloff J.J., Garcia-Ocaña A. (2004). Prevention of acute ischemic renal failure by targeted delivery of growth factors to the proximal tubule in transgenic mice: the efficacy of parathyroid hormone-related protein and hepatocyte growth factor. J. Am. Soc. Nephrol..

[50] Wang H., Li Y., Zheng G. (2026). BMP7 in kidney development and disease: a multilayer regulatory network integrating inflammation, fibrosis, and regeneration. Cell Signal. Inflamm. Dis..

[51] Nagayama I., Takei Y., Takahashi S., Okada M., Maeshima A. (2025). The activin-follistatin system: Key regulator of kidney development, regeneration, inflammation, and fibrosis. Cytokine Growth Factor Rev..

[52] Nagayama I., Takayanagi K., Hasegawa H., Maeshima A. (2023). Tubule-Derived Follistatin Is Increased in the Urine of Rats with Renal Ischemia and Reflects the Severity of Acute Tubular Damage. Cells.

[53] Chiba T., Skrypnyk N.I., Skvarca L.B., Penchev R., Zhang K.X., Rochon E.R., Fall J.L., Paueksakon P., Yang H., Alford C.E. (2016). Retinoic Acid Signaling Coordinates Macrophage-Dependent Injury and Repair after AKI. J. Am. Soc. Nephrol..

[54] Isabella A.J., Barsh G.R., Stonick J.A., Dubrulle J., Moens C.B. (2020). Retinoic Acid Organizes the Zebrafish Vagus Motor Topographic Map via Spatiotemporal Coordination of Hgf/Met Signaling. Dev. Cell.

[55] Issa R.S., Kaehler M., Pommert N.S., Cascorbi I., Waetzig V. (2024). Enhancing Retinoic Acid-mediated Effects Through Inhibition of CYP26A1, CYP26B1 and HGF Signaling in Neuroblastoma Cells. Anticancer Res..

[56] Paralkar V.M., Grasser W.A., Mansolf A.L., Baumann A.P., Owen T.A., Smock S.L., Martinovic S., Borovecki F., Vukicevic S., Ke H.Z., Thompson D.D. (2002). Regulation of BMP-7 expression by retinoic acid and prostaglandin E(2). J. Cell. Physiol..

[57] Tsuji K., Kitamura S., Wada J. (2022). Potential Strategies for Kidney Regeneration With Stem Cells: An Overview. Front. Cell Dev. Biol..

[58] Liu J., Hu J., Gao X., Liu H. (2025). Twenty-year panoramic review of stem cells research in kidney diseases (2005-2025): a bibliometric and clinical trial-based exploration. Int. J. Surg..

[59] Lerman L.O. (2021). Cell-based regenerative medicine for renovascular disease. Trends Mol. Med..

[60] Han X., Sun Z. (2022). Adult Mouse Kidney Stem Cells Orchestrate the De Novo Assembly of a Nephron via Sirt2-Modulated Canonical Wnt/β-Catenin Signaling. Adv. Sci..

[61] Mu L., Dong R., Guo B. (2023). Biomaterials-Based Cell Therapy for Myocardial Tissue Regeneration. Adv. Healthc. Mater..

[62] Hickson L.J., Abedalqader T., Ben-Bernard G., Mondy J.M., Bian X., Conley S.M., Zhu X., Herrmann S.M., Kukla A., Lorenz E.C. (2021). A systematic review and meta-analysis of cell-based interventions in experimental diabetic kidney disease. Stem Cells Transl. Med..

[63] Montenegro F., Giannuzzi F., Picerno A., Cicirelli A., Stea E.D., Di Leo V., Sallustio F. (2024). How Stem and Progenitor Cells Can Affect Renal Diseases. Cells.

[64] Liu P., Mao Y., Xie Y., Wei J., Yao J. (2022). Stem cells for treatment of liver fibrosis/cirrhosis: clinical progress and therapeutic potential. Stem Cell Res. Ther..

[65] Huang J., Kong Y., Xie C., Zhou L. (2021). Stem/progenitor cell in kidney: characteristics, homing, coordination, and maintenance. Stem Cell Res. Ther..

[66] Zhang Y., Wang X., Ji Y., Hong H., Geng X., Zhang K., Fu Z., Cai G., Chen X., Li P., Hong Q. (2024). All-trans retinoic acid pretreatment of mesenchymal stem cells enhances the therapeutic effect on acute kidney injury. Cell Commun. Signal..

[67] Barakat M., Hussein A.M., Salama M.F., Awadalla A., Barakat N., Serria M., El-Shafey M., El-Sherbiny M., El Adl M.A. (2022). Possible Underlying Mechanisms for the Renoprotective Effect of Retinoic Acid-Pretreated Wharton's Jelly Mesenchymal Stem Cells against Renal Ischemia/Reperfusion Injury. Cells.

[68] Hejazian S.M., Hejazian S.S., Mostafavi S.M., Hosseiniyan S.M., Montazersaheb S., Ardalan M., Zununi Vahed S., Barzegari A. (2024). Targeting cellular senescence in kidney diseases and aging: A focus on mesenchymal stem cells and their paracrine factors. Cell Commun. Signal..

[69] Hong X., Nie H., Deng J., Liang S., Chen L., Li J., Gong S., Wang G., Zuo W., Hou F., Zhang F. (2023). WT1(+) glomerular parietal epithelial progenitors promote renal proximal tubule regeneration after severe acute kidney injury. Theranostics.

[70] Peired A.J., Melica M.E., Molli A., Nardi C., Romagnani P., Lasagni L. (2021). Molecular Mechanisms of Renal Progenitor Regulation: How Many Pieces in the Puzzle?. Cells.

[71] Kazeminia S., Eirin A. (2024). Role of mitochondria in endogenous renal repair. Clin. Sci..

[72] Ciarambino T., Crispino P., Giordano M. (2022). Gender and Renal Insufficiency: Opportunities for Their Therapeutic Management?. Cells.

[73] Wang M., Wu W., Lu J., Lu R., Min L., Song A., Zhao B., Li Y., Xie K., Gu L. (2025). Mitochondrial-Derived Signaling Mediates Differentiation of Parietal Epithelial Cells into Podocytes. Antioxid. Redox Signal..

[74] Dai Y., Chen A., Liu R., Gu L., Sharma S., Cai W., Salem F., Salant D.J., Pippin J.W., Shankland S.J. (2017). Retinoic acid improves nephrotoxic serum-induced glomerulonephritis through activation of podocyte retinoic acid receptor α. Kidney Int..

[75] Liu W.B., Fermin D., Xu A.L., Kopp J.B., Xu Q. (2024). Single-cell RNA sequencing data locate ALDH1A2-mediated retinoic acid synthetic pathway to glomerular parietal epithelial cells. Exp. Biol. Med..

[76] Peired A., Angelotti M.L., Ronconi E., la Marca G., Mazzinghi B., Sisti A., Lombardi D., Giocaliere E., Della Bona M., Villanelli F. (2013). Proteinuria impairs podocyte regeneration by sequestering retinoic acid. J. Am. Soc. Nephrol..

[77] Tsujimoto H., Hoshina A., Mae S.-I., Araoka T., Changting W., Ijiri Y., Nakajima-Koyama M., Sakurai S., Okita K., Mizuta K. (2024). Selective induction of human renal interstitial progenitor-like cell lineages from iPSCs reveals development of mesangial and EPO-producing cells. Cell Rep..

[78] Kurtzeborn K., El-Dahr S.S., Pakkasjärvi N., Tortelote G.G., Kuure S. (2025). Kidney development at a glance: metabolic regulation of renal progenitor cells. Curr. Top. Dev. Biol..

[79] Sanchez-Ferras O., Pacis A., Sotiropoulou M., Zhang Y., Wang Y.C., Bourgey M., Bourque G., Ragoussis J., Bouchard M. (2021). A coordinated progression of progenitor cell states initiates urinary tract development. Nat. Commun..

[80] Wang C., Spradling A.C. (2022). Drosophila renal stem cells enhance fitness by delayed remodeling of adult Malpighian tubules. Sci. Adv..

[81] Sierra-Mondragon E., Rodríguez-Muñoz R., Namorado-Tonix C., Molina-Jijon E., Romero-Trejo D., Pedraza-Chaverri J., Reyes J.L. (2019). All-Trans Retinoic Acid Attenuates Fibrotic Processes by Downregulating TGF-β1/Smad3 in Early Diabetic Nephropathy. Biomolecules.

[82] Zhu J., Wang M., Zhang Y., Wang X., Dai L., Li Z., Li Y., Zhang X., Wang H., Zhao J. (2026). Renal tubular-derived retinoic acid drives renal fibrosis via a RAI14-TRIOBP-YAP mechanotransduction axis. Cell. Signal..

[83] Chen A., Liu Y., Lu Y., Lee K., He J.C. (2021). Disparate roles of retinoid acid signaling molecules in kidney disease. Am. J. Physiol. Renal Physiol..

[84] Feng Y., Sun Z., Fu J., Zhong F., Zhang W., Wei C., Chen A., Liu B.C., He J.C., Lee K. (2024). Podocyte-derived soluble RARRES1 drives kidney disease progression through direct podocyte and proximal tubular injury. Kidney Int..

[85] Medina Rangel P.X., Ishibe S. (2024). Preventing MMP23-mediated cleavage of podocyte RARRES1: a novel strategy to halt chronic kidney disease progression?. Kidney Int..

[86] Chen A., Feng Y., Lai H., Ju W., Li Z., Li Y., Wang A., Hong Q., Zhong F., Wei C. (2020). Soluble RARRES1 induces podocyte apoptosis to promote glomerular disease progression. J. Clin. Investig..

[87] Ferreira R., Napoli J., Enver T., Bernardino L., Ferreira L. (2020). Advances and challenges in retinoid delivery systems in regenerative and therapeutic medicine. Nat. Commun..

[88] Cristiano M.C., Cosco D., Celia C., Tudose A., Mare R., Paolino D., Fresta M. (2017). Anticancer activity of all-trans retinoic acid-loaded liposomes on human thyroid carcinoma cells. Colloids Surf. B Biointerfaces.

[89] Choi E.J., Kim N., Kwak H.-S., Han H.J., Chun K.-C., Kim Y.-A., Koh J.-W., Han J.Y., Joo S.H., Lee J.S., Koren G. (2021). The rates of major malformations after gestational exposure to isotretinoin: a systematic review and meta-analysis. Obstet. Gynecol. Sci..

[90] Hu P., van Dam A.M., Wang Y., Lucassen P.J., Zhou J.N. (2020). Retinoic acid and depressive disorders: Evidence and possible neurobiological mechanisms. Neurosci. Biobehav. Rev..

[91] Huang H., Lu W., Luo R., Zeng Y., Zhang Y., Su X., Zhang X., Tian B., Wang X. (2024). Astrocytic RARγ mediates hippocampal astrocytosis and neurogenesis deficits in chronic retinoic acid-induced depression. Neuropsychopharmacology.

[92] Asfour M.H., Salama A.A.A., Mohsen A.M. (2021). Fabrication of All-Trans Retinoic Acid loaded Chitosan/Tripolyphosphate Lipid Hybrid Nanoparticles as a Novel Oral Delivery Approach for Management of Diabetic Nephropathy in Rats. J. Pharm. Sci..

[93] Huang Y., Wang J., Jiang K., Chung E.J. (2021). Improving kidney targeting: The influence of nanoparticle physicochemical properties on kidney interactions. J. Control. Release.

[94] Cheng H.T., Ngoc Ta Y.N., Hsia T., Chen Y. (2024). A quantitative review of nanotechnology-based therapeutics for kidney diseases. Wiley Interdiscip. Rev. Nanomed. Nanobiotechnol..

[95] Wang X., Lin Y., Zeng Y., Sun X., Gong T., Zhang Z. (2013). Effects of mycophenolic acid-glucosamine conjugates on the base of kidney targeted drug delivery. Int. J. Pharm..

[96] Huang C., Zeng T., Li J., Tan L., Deng X., Pan Y., Chen Q., Li A., Hu J. (2019). Folate Receptor-Mediated Renal-Targeting Nanoplatform for the Specific Delivery of Triptolide to Treat Renal Ischemia/Reperfusion Injury. ACS Biomater. Sci. Eng..

[97] He S., Li X., He Y., Guo L., Dong Y., Wang L., Yang L., Li L., Huang S., Fu J. (2025). High-density lipoprotein nanoparticles spontaneously target to damaged renal tubules and alleviate renal fibrosis by remodeling the fibrotic niches. Nat. Commun..

[98] Wischnjow A., Sarko D., Janzer M., Kaufman C., Beijer B., Brings S., Haberkorn U., Larbig G., Kübelbeck A., Mier W. (2016). Renal Targeting: Peptide-Based Drug Delivery to Proximal Tubule Cells. Bioconjug. Chem..

[99] He J., Chen H., Zhou W., Chen M., Yao Y., Zhang Z., Tan N. (2020). Kidney targeted delivery of asiatic acid using a FITC labeled renal tubular-targeting peptide modified PLGA-PEG system. Int. J. Pharm..

[100] Pan X., Xie F., Xiao D., Zhou X., Xiao J. (2020). Design, Synthesis, and Renal Targeting of Methylprednisolone-Lysozyme. Int. J. Mol. Sci..

[101] Xiao Y., Tong Z., Xu H., Jia Z., Wang C., Cao Y., Song L., Hao S., Yang J., Zhou Y. (2025). A rationally designed injury kidney targeting peptide library and its application in rescuing acute kidney injury. Sci. Adv..

[102] Rhim W.K., Woo J., Kim J.Y., Lee E.H., Cha S.G., Kim D.S., Baek S.W., Park C.G., Kim B.S., Kwon T.G., Han D.K. (2025). Multiplexed PLGA scaffolds with nitric oxide-releasing zinc oxide and melatonin-modulated extracellular vesicles for severe chronic kidney disease. J. Adv. Res..

[103] Zhang F., Zhang A., Xie Y., Wen H., Kankala R.K., Huang J., Zhang A., Wang Q., Chen B., Dong H. (2023). Nanocarrier of Pin1 inhibitor based on supercritical fluid technology inhibits cancer metastasis by blocking multiple signaling pathways. Regen. Biomater..

[104] Jeon C., Jun H., Kim S., Song N., Yang M., Lim C., Lee D. (2023). Clot-Targeted Antithrombotic Liposomal Nanomedicine Containing High Content of H(2)O(2)-Activatable Hybrid Prodrugs. ACS Appl. Mater. Interfaces.

[105] Lee S.J., Cho J.Y., Heo T.H., Yang D.H., Chun H.J., Yoon J.K., Jeong G.J. (2025). Recent advances in applications of nanoparticles and decellularized ECM for organoid engineering. Mater. Today Bio.

[106] Boo Y., Choi S.H., Kim J., Kim W.J. (2025). Nitric oxide-modulating biomaterials for therapeutic Immunoengineering. Adv. Drug Deliv. Rev..

[107] Greening D.W., Xu R., Ale A., Hagemeyer C.E., Chen W. (2023). Extracellular vesicles as next generation immunotherapeutics. Semin. Cancer Biol..

[108] Putthanbut N., Lee J.Y., Borlongan C.V. (2024). Extracellular vesicle therapy in neurological disorders. J. Biomed. Sci..

[109] Wu Z., Kuang Z., Liang L., Liu J., Wang G., Zou F. (2026). TGF-β/LAMB3 axis drives ROS-dependent renal fibrosis under hypoxic conditions. Free Radic. Biol. Med..

[110] Murphy N.P., Lampe K.J. (2018). Fabricating PLGA microparticles with high loads of the small molecule antioxidant N-acetylcysteine that rescue oligodendrocyte progenitor cells from oxidative stress. Biotechnol. Bioeng..

[111] Lenas P., Ikonomou L. (2018). Developmental engineering: design of clinically efficacious bioartificial tissues through developmental and systems biology. Sci. China Life Sci..

[112] Lenas P. (2018). Developmental biology in bioartificial tissue design: manufacturing and regulatory considerations. Regen. Med..

[113] Daly A.C., Cunniffe G.M., Sathy B.N., Jeon O., Alsberg E., Kelly D.J. (2016). 3D Bioprinting of Developmentally Inspired Templates for Whole Bone Organ Engineering. Adv. Healthc. Mater..

[114] Qin Z., Cao L., Liu C., An Y., Li F., Yang X., Chen G., Qian M., Zhang Y., Yang J. (2026). Cadherin-engineered microspheres enable scalable biofabrication of multilineage liver organoids. Trends Biotechnol..

[115] Schnell J., Miao Z., Achieng M., Fausto C.C., Koppitch K., Takhirov L., Wang V., De Kuyper F., Huang B., Schreiber M. (2025). Controlling nephron precursor differentiation to generate proximal-biased kidney organoids with emerging maturity. Nat. Commun..

[116] Little M.H. (2021). Returning to kidney development to deliver synthetic kidneys. Dev. Biol..

[117] Little M.H., McMahon A.P. (2012). Mammalian kidney development: principles, progress, and projections. Cold Spring Harb. Perspect. Biol..

[118] Xu L., Feng Z., Sinha D., Ducos B., Ebenstein Y., Tadmor A.D., Gauron C., Le Saux T., Lin S., Weiss S. (2012). Spatiotemporal manipulation of retinoic acid activity in zebrafish hindbrain development via photo-isomerization. Development.

[119] Nolte T.M., Lu B. (2024). Size and dose of nanoparticles modulate between toxic and medicinal effect on kidney. OpenNano.

[120] Du B., Yu M., Zheng J. (2018). Transport and interactions of nanoparticles in the kidneys. Nat. Rev. Mater..

